# Application of array comparative genomic hybridization (aCGH) for identification of chromosomal aberrations in the recurrent pregnancy loss

**DOI:** 10.1007/s10815-022-02400-8

**Published:** 2022-01-26

**Authors:** Katarzyna Kowalczyk, Marta Smyk, Magdalena Bartnik-Głaska, Izabela Plaskota, Barbara Wiśniowiecka-Kowalnik, Joanna Bernaciak, Marta Chojnacka, Magdalena Paczkowska, Magdalena Niemiec, Daria Dutkiewicz, Agata Kozar, Róża Magdziak, Wojciech Krawczyk, Grzegorz Pietras, Elżbieta Michalak, Teresa Klepacka, Ewa Obersztyn, Jerzy Bal, Beata Anna Nowakowska

**Affiliations:** 1 Department of Medical Genetics, Institute of Mother and Child, Warsaw, Poland; 2 Department of Obstetrics and Perinatology, Medical University of Lublin, Lublin, Poland; 3 Deparment of Pathomorphology, Institute of Mother and Child, Warsaw, Poland

**Keywords:** Microarray, Chromosomal aberrations, Spontaneous abortion

## Abstract

Spontaneous abortion occurs in 8–20% of recognized pregnancies and usually takes place in the first trimester (7–11 weeks). There are many causes of pregnancy loss, but the most important (about 75%) is the presence of chromosomal aberrations. We present the results of oligonucleotide array application in a cohort of 62 miscarriage cases. The inclusion criteria for the study were the loss after 8th week of pregnancy and the appearance of recurrent miscarriages. DNA was extracted from trophoblast or fetal skin fibroblasts. In the 62 tested materials from recurrent miscarriages, the detection rate was 56.5% (35/62). The most commonly found were aneuploidies (65%) (chromosomal trisomy 14, 16, 18, 21, and 22), Turner syndrome, and triploidy (17.1%). Other chromosomal abnormalities included pathogenic and likely pathogenic structural aberrations: 1) pathogenic: deletion 7p22.3p12.3 and duplication 9p24.3p13.2 inherited from the normal father, deletion 3q13.31q22.2 and deletion 3q22.3q23 of unknown inheritance and duplication of 17p12 inherited from father with foot malformation; 2) likely pathogenic variants: deletion 17p13.1 inherited from normal mother, deletion 5q14.3 of unknown inheritance and de novo deletion 1q21.1q21.2. Among these aberrations, six CNVs (copy number variants) were responsible for the miscarriage: deletion 7p22.3p12.3 and duplication 9p24.3p13.2, deletion 3q13.31q22.2 and deletion 3q22.3q23, and deletion 17p13.1 and deletion 1q21.1q21.2. Other two findings were classified as incidental findings (deletion 5q14.3 and 17p12 duplication). Our research shows that 17% of the aberrations (6/35 abnormal results) that cannot be identified by the routine kariotype analysis are structural aberrations containing genes important for fetal development, the mutations of which may cause spontaneous abortion.

## Introduction 

The process of reproduction is conditioned by many factors: genetic, epigenetic, and environmental. The couples who are facing recurrent pregnancy failure must be prepared for lengthy diagnostic process that does not always lead to a clear answer on the cause of miscarriage. Most frequently cited causes of failures of pregnancy are as follows: endocrine disorders, autoimmune diseases, metabolic diseases, reproductive system anatomical defects, and genetic diseases (El Hachem et al. 2017). Anatomical defects of the reproductive system and in particular the uterus may increase the risk of pregnancy loss in the first or second trimester. Anatomical uterine abnormalities are identified in 10–15% of recurrent miscarriages. Equally important reason for miscarriages is caused by hormonal disorders such as excessive secretion of LH, hyperprolactinemia, high levels of androgens, and polycystic ovary syndrome (Berghella 2007). It is estimated that 8–20% of recognized pregnancies spontaneous abortion occur in the first trimester (7–11 weeks) (Bug et al. 2014).

Among the many causes of pregnancy loss, the most important group (approximately 75%) are fetal/embryo chromosomal aberrations (El Hachem et al. 2017). This high level of chromosomal aberration is due to an abnormality in the genetic material of reproductive cells. Previous studies indicate that 20–30% of eggs in women, and 6–8% of sperm in young, healthy, fertile men showed a chromosomal aberration (most often it is an abnormal number of chromosomes) (Caseiro et al. 2015, Gao et al. 2012, ESHRE 2008, Simpson 2007, Zhou et al. 2016).

Genetic research on pregnancy failure dates back to the second half of the twentieth century, just after the normal number of chromosomes in the human cell nucleus was established in 1956 by Joe Hin Tijo and Johan Albert Levan (Lawce and Brown 1997). With advances in cytogenetic techniques and conducting long-term research into the genetic causes of pregnancy failure, chromosome aberrations were discovered as a major cause of pregnancy loss (Carr 1971).

Before the era of molecular cytogenetic, a routine kariotype analysis by GTG techniquewas the only method used to detect trisomy and monosomy of whole chromosomes or deletions and duplications greater than 5–10 Mb in size. Over the past several years, new techniques for fast diagnosis of the most common chromosomal aneuploidy in the fetus were introduced. They allow to significantly reduce the waiting time for the result (24–48 h), reduce the cost of the test, and reduce the workload. These techniques include the following: Rapid-FISH (rapid fluorescent in situ hybridization), which is performed on non-cultured cell nuclei, isolated immediately after the collection of abortive material. Others, such as QF-PCR (quantitative fluorescent polymerase chain reaction), BoBs (BACs-on beads), MLPA (multiplex ligation-dependent probe amplification) technique, and CGH microarray (comparative genomic hybridization), are performed on DNA isolated from uncultured cells. The first scientific report on the array CGH method in pregnancy failure was published in 2004 and showed that this method with high resolution enables the detection of unbalanced aberrations in the genome (Schaeffer 2004). For the next few years, it was proven that the aCGH (array-CGH) is the most effective and quickest method for detecting chromosomal aberrations in the material of miscarriage. It is the only technology enabling the identification of all unbalanced aberrations (number and structure) with a much higher resolution than the usually applied classical karyotype.

Previous research using microarrays showed that ~ 42% chromosomal aberrations detected in aborted spontaneously embryos/fetuses are trisomies (mainly chromosomes 16, 18, and 22), which occurs mostly as a result of errors during meiosis I (Pellestor et al. 2005; van den Berg et al. 2012; Berghella et al. 2007). Other frequently detected chromosomal aberrations are monosomy X (approx. 10–18% of miscarriages), chromosomal triploidy, and chromosomal tetraploidy (including about 10% of miscarriages) (Viaggi et al. 2013; Dória et al. 2009). The incidence of structural aberrations is typically in the range of 0 to 9% (Berghella et al. 2007). Examples of the recurrent structural aberrations responsible for miscarriages are as follows: deletions (the most common: 1p36.13; 2p11.2, 3q29, 13q12.11) or duplications (the most common: 8q12.1; 15q11.11q11.12; Xq22.2). Zhou et al. (2016); Viaggi et al. (2013); Shimokawa et al. (2006); Shaeffer et al. (2004); Pertile et al. (2012); and Shen et al. (2016) have analyzed over 2,000 cases of pregnancy failure (spontaneous abortions, stillbirths, and fetal birth defects) using the CGH method. They achieved results in 98% of cases, with the most common chromosomal aberrations in the examined material were aneuploidy (Pertile et al. 2012). In addition to numerical aberrations, also microdeletion/microduplication syndromes, such as Williams syndrome (deletion 7q11.23), duplication 7q11.23, Angelman or Prader-Willi syndrome (deletion 15q11.2q13), DiGeorge syndrome (deletion 22q11.21), and Sotos syndrome (deletion 5q35.3), were detected. Moreover, CNVs (copy number variants) of unknown clinical significance were identified in 4.1% of cases (Pertile et al. 2012).

In our study, we identified pathogenic and likely pathogenic aberrations in 35 out of 62 (56.5%) miscarriages. Chromosomal aneuploidy was detected in 23 cases (65.8%, 23/35), structural aberrations in 6 cases (17.1%, 6/35), 5 cases (14.3%, 5/35) with chromosomal polyploidy, and 1 case (2.8%, 1/35) with mosaic tetrasomy with extra pair of chromosome 13.

## Materials and methods

We received samples of biological material from miscarriages and their parents after signing the informed consent, using protocols accepted by the Bioethics Committee at the Institute of Mother and Child in Warsaw (opinion number: 35/2017).

### Sample collection

**Fig. 1:**
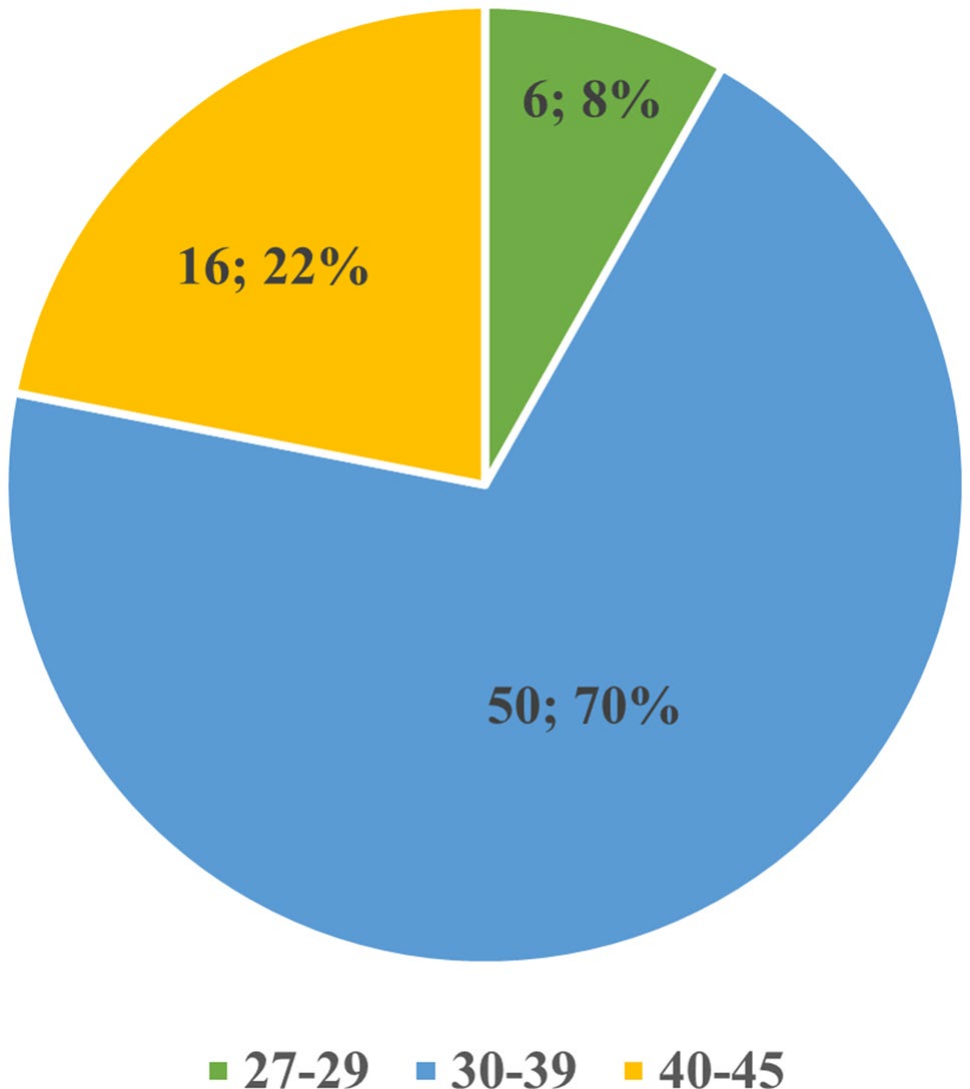
Number of patients’ age groups: 6 patients between 27 and 29 years old, 50 patients in the range of 30–39 years old, 16 patients between 40 and 45 years old

**Fig. 2:**
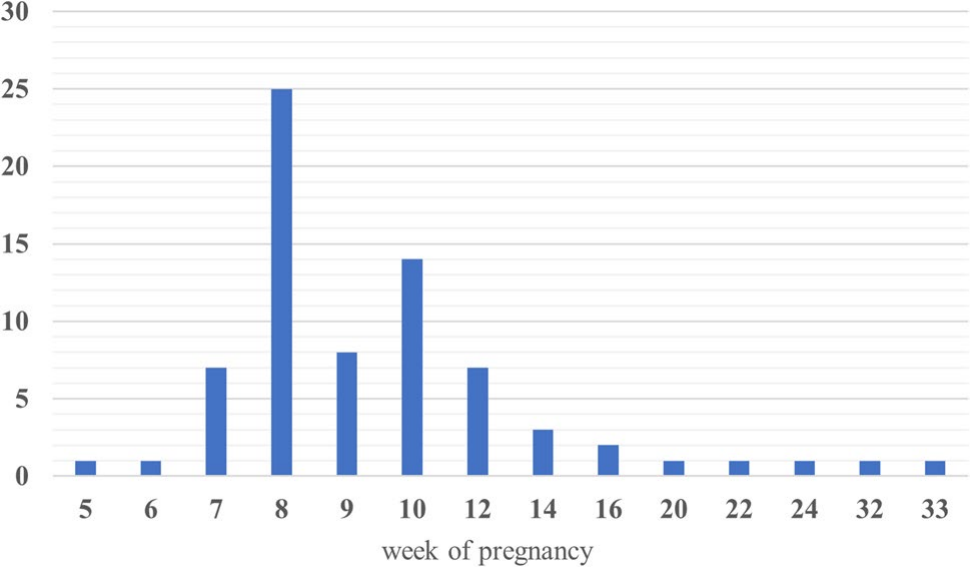
Number of miscarriages in individual weeks of pregnancy

Seventy-two patients were qualified for the project. Patients aged 30–39 (Fig. 1). The mean age of the patients and the median were 36 years. The average time of miscarriage occurred in the 8th week of pregnancy (Fig. 2).

Samples were received by the Cytogenetics Laboratory of the Institute of Mother and Child in Warsaw for over a 3-year period from ten obstetrics centers from Poland. The indication for the study was at least third recurrent spontaneous abortion. The obtained biological material was subjected to histopathological assessment at the Pathomorphology Department, Institute of Mother and Child. Ethical approval was granted for this study in all hospitals.

Fifty women were admitted at third miscarriage, 13 at fourth miscarriage, 7 at fifth miscarriage, 1 at sixth miscarriage, and 1 at twelfth miscarriage. Miscarriages occurred in the first, second, and third trimester. None of the women had metabolic, autoimmune, or other systemic disorders or uterine anatomic abnormalities.

Eleven products of conception were excluded from the further analysis because the fetal material was not found. The aCGH test was performed on 61 cases; however, one patient reported twin pregnancy, so we investigated 62 products of conception.

### Sample types and DNA isolation

Forty-seven trophoblast and 15 fetal skin fibroblasts samples were used for our study. Genomic DNA was immediately isolated from fresh material. Villi from trophoblast were separated from maternal tissue under a microscope to minimize maternal cell contamination (MCC). Two to four villi were provided for DNA extraction. Genomic DNA was extracted using the DNA isolation kit (Sherlock A&A Biotechnology, Poland) following the manufacturers recommendations. For trophoblast and skin fibroblasts, incubation at 56 °C with 20 µl proteinase K, water, and tissue lysis buffer (Buffer L1.4) was performed for at least 1 h for efficient digestion and lysis of the complete sample. DNA isolation was performed according to the manufacturer’s instruction.

### Genomic array platform (array comparative genomic hybridization (array CGH) analysis and interpretation): method and analysis

Array CGH was performed using 4 × 180 K microarrays from Oxford Gene Technology (CytoSure ISCA, v3, Oxford, UK). The array used in this study contains 60-mer oligonucleotides probes covering the whole genome with an average spatial resolution of 24 kb. Description of aCGH methodology is available in the Supplementary Material online. All genomic coordinates are based on reference genome (NCBI37/hg19). Data analysis was performed using the CytoSure Interpret Software (Oxford Gene Technology, Oxford, UK) and the circular binary segmentation algorithm. The calling thresholds were deviation of a circular binary segmentation (CBS) segment from zero log ratio of + 0.30 for duplications and − 0.5 for deletions. Results were then classified with CytoSure Interpret Software (Oxford Gene Technology, Oxford, UK). Quality control metrics are monitored with CytoSure Interpret Software (Oxford Gene Technology).

The microarray used in this analysis does not contain SNP probes, and it does not detect polyploidy, inversion, balanced translocation, and regions of absence of heterozygosity. In the case of polyploidy, using the CGH method, we can only suspect its presence in male fetus when calling thresholds were deviation of a circular binary segmentation (CBS) segment from zero log ratio of + 0.2 for chromosome X and − 0.4 for chromosome Y.

#### CNV classification

The clinical relevance of copy number of variants should be considered individually using the general CNV classification. In our study, we used five categories classification of results: pathogenic, likely pathogenic, variants of unknown significance (VOUS), likely benign, and benign:

1. Pathogenic aberrations
  CNVs of several Mb in size (generally > 5 Mb), or it is one of the recurrent genomic disorders and known microdeletion/microduplication syndromes, or it is containing known genes involved in a particular pathology and the CNV which was previously described in specific clinical disorders.
2. Likely pathogenic aberrations
  CNVs were previously described in another patient with recurrent miscarriages or contain some gene/genes whose function is known and may be responsible for birth defects and/or fetal death.
3. Variants of unknown significance (VOUS)
  This category includes all CNVs that have no clearly defined clinical relevance at the time the test result is published. These aberrations were not reported in our results, because the function of genes in this region is unknown or difficult to associate with the recurrent fetal loss.
4. Likely benign aberrations
  CNVs that have not been described but are present in healthy parents and have only been described in a few cases in the general population, but do not represent a common polymorphism. CNVs interpreted as likely benign were not reported.
5. Benign aberrations
  CNVs which do not affect the phenotype (found in the general population), which include aberrations in the region of segmental duplication, aberrations which do not contain genes, aberrations in areas containing dose-insensitive genes often recurring in the Polish population, and aberrations described in the Database of Genomic Variants database (http://dgv.tcag.ca/dgv/app/home ) (track: DGV Gold Standard Variants). Benign CNVs were not reported.

Detected copy number variants were systematically evaluated for clinical significance by comparing them with those in the scientific literature and available databases: OMIM (http://www.ncbi.nlm.nih.gov/omim), ISCA (https://www.iscaconsortium.org/), Database of Genomic Variants (http://projects.tcag.ca/variation/), Ensembl (https://www.ensembl.org/index.html), and DECIPHER (http://decipher.sanger.ac.uk/). In our research, we reported pathogenic and likely pathogenic aberrations.

### Rapid-FISH: method and analysis

The second method used in the project was Rapid-FISH. This technique was used to exclude chromosomal polyploidy in aCGH results with normal female hybridization pattern. Additionally, we used these methods when we suspected presence of chromosomal polyploidy in the male fetus based on the CGH result. Rapid-FISH involves the hybridization of a fluorescently labeled genetic probe with selected sequences of a given chromosome. This method is performed on interphase nuclei immediately after collecting a small amount of trophoblast, which allows obtaining the result of a genetic test within 2–5 days. The analysis of Rapid-FISH results is based on the number assessment selected chromosomes after the use of molecular probes. We used commercially available set of probes contained centromeric probes for X, Y, 16, and 18 chromosomes and probes specific for critical regions of chromosomes 13, 21, and 22 (CytoCell Aquarius or Vysis).

The Rapid-FISH test was performed using a CytoCell Aquarius or Vysis probe kit according to the attached procedure. For fluorescence analysis, we used a Nikon Eclipse E400 (USA) microscope. For each preparation, at least 30 interphase nuclei were analyzed, and if mosaicism was suspected, 100 nuclei were analyzed.

## Results

Normal aCGH results were obtained in 27 samples (43.5%). In 22 female fetuses with normal CGH results, Rapid-FISH was carried out to exclude chromosomal polyploidy. In 3 cases (3/22, 13.6%) with the normal aCGH test result, the Rapid-FISH test showed chromosomal triploidy. One patient had normal aCGH result, while Rapid-FISH found mosaic trisomy of chromosome 13 in 9% cells (1/22, 4.5%).

**Fig. 3:**
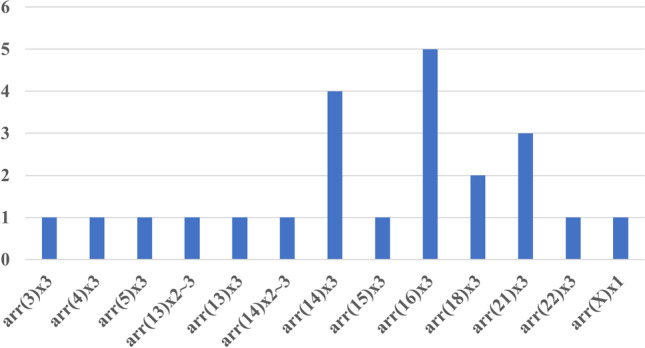
Number of cases with chromosomal aneuploidy in all 35 abnormal results (arr(…)×3 trisomy of chromosome, arr(14)×2~3 mosaic trisomy of chromosome 14, arr(X)×1 monosomy of chromosome X)

**Fig. 4:**
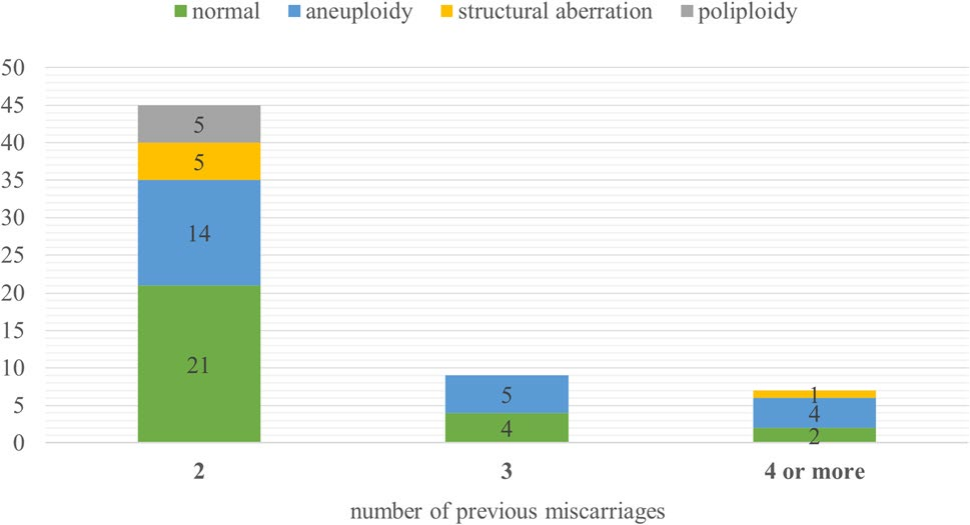
Number of cases with normal results, aneuploidy, polyploidy, and structural aberration in three subgroups of different number of previous miscarriages

**Fig. 5:**
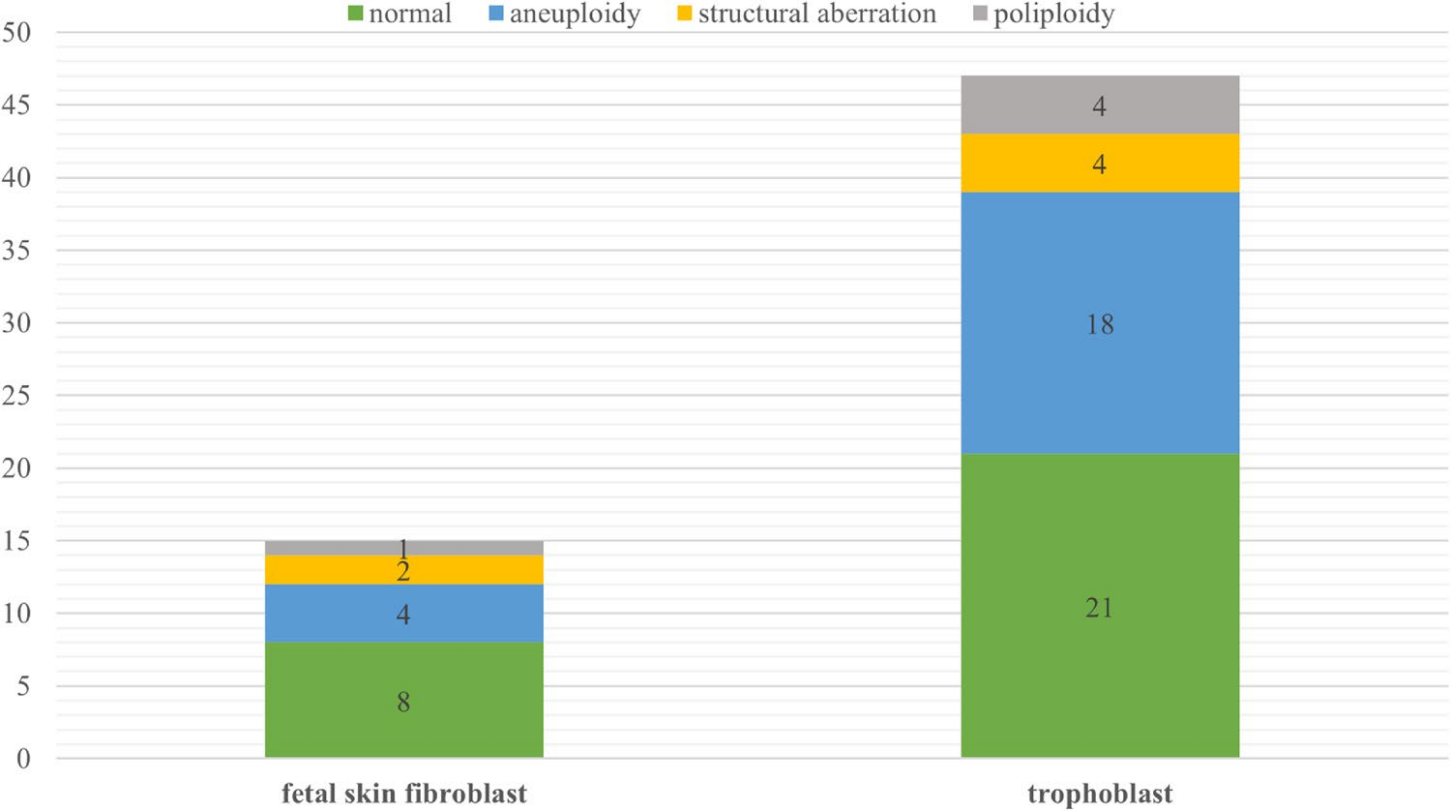
Number of cases with normal results, aneuploidy, polyploidy, and structural aberration in two subgroups of different material for DNA extraction

Abnormal aCGH and Rapid-FISH results were found in 35 cases (56.5% of cases). Chromosomal aneuploidy was detected in 23 cases (Table 1). Pathogenic structural aberrations were found in 3 cases and likely pathogenic in 3 cases. Aberrations responsible for miscarriage include the following: deletion 7p22.3p12.3 and duplication 9p24.3p13.2 (derived from parental balanced translocation), deletion 3q13.31q22.2 and deletion 3q22.3q23, and deletion 17p13.1 and deletion 1q21.1q21.2 (Table 1). The most common aneuploidy is chromosomal trisomy 14 and 16 (Fig. 3). Additionally, we detected 5 cases with chromosomal polyploidy (Table 1) and 1 case with mosaic tetrasomy with extra pair of chromosome 13. In the subgroup of patients with 2 previous spontaneous abortions, the most numerous finding was aneuploidy; in the subgroup of 3 previous miscarriages, we found only aneuploidy; and in the subgroup of 4 or more miscarriages, the aberration detection rate was the highest among all three groups (5 abnormal results to 2 normal results, 71.4%) (Fig. 4). In two subgroup of different material for extraction DNA (fetal skin fibroblast and trophoblast), we did not notice any significant difference between normal and abnormal results. In both subgroups, the percentage of abnormal findings was approximately 50% (46% in fetal skin fibroblast and 55% in trophoblast) Fig. 5.

**Table 1:**
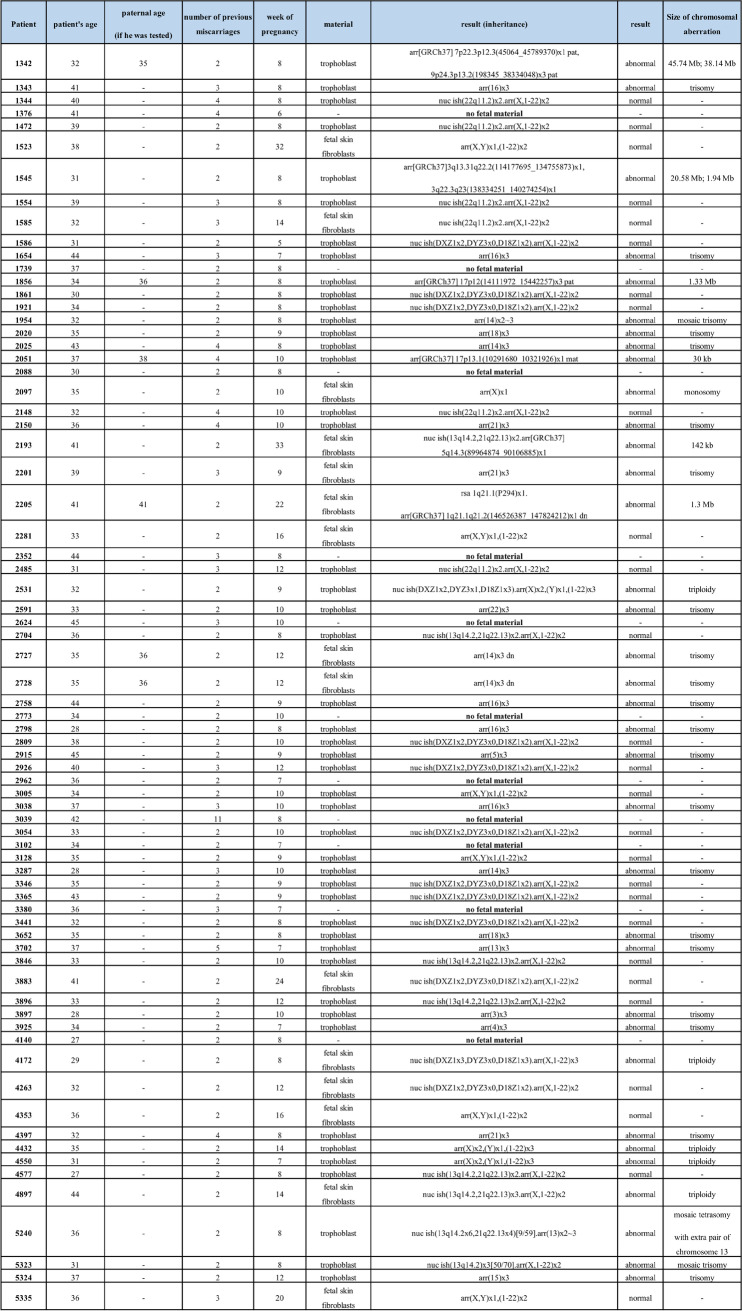
Summary of the results, taking into account the age of the patient and the father, the number of previous miscarriages, the week of pregnancy loss, and the type of material tested

## Discussion

Spontaneous miscarriage is a very difficult experience for a couple expecting a child, and the uncertainty as to the cause of the miscarriage and the fear that subsequent pregnancies may also fail are inextricably linked with this dramatic event. Determining the cause of a miscarriage makes it possible to predict the chances of sustaining future pregnancies and indicates the direction of therapeutic measures, and for a couple who experienced pregnancy loss, it is of great psychological importance. Of all the causes of spontaneous miscarriages, genetic factors are the most important, therefore, genetic testing is an indispensable part of the diagnosis of couples with miscarriages. These tests may also indicate an increased genetic risk of having a baby with a serious genetic condition.

Technologies based on microarray methods such as the CGH array, sometimes referred to as “molecular karyotype,” allow the identification of the whole spectrum of CNV sizes, starting from aneuploidy and ending on very small submicroscopic aberrations. Therefore, a microarray method is recommended as a diagnostic test for the fetus with abnormal ultrasound result, but also in post-miscarriage chorionic villus testing (Miller et al. 2010).

For proper recognition of identified CNV and estimation of its impact on the prognosis of pregnancy, several databases are publicly available. The most commonly used are as follows: ClinGen (https://clinicalgenome.org/), DECIPHER (https://www.deciphergenomics.org/), and Database of Genomic Variants (DGV) (http://dgv.tcag.ca/dgv/app/home). In most research reports, CNVs were classified into five groups: benign, likely benign, variants of unknown significance, likely pathogenic, and pathogenic, in line with the European Clinical Genetics laboratories (Silva et al. 2019) and the ACMG Standards and Guidelines (South et al. 2013). Additionally, parental testing was performed in cases where unbalanced structural rearrangements were identified, in order to possibly confirm their origin.

In our study, in 56.5% of cases (35 out of 62 samples) obtained after pregnancy failures, chromosomal abnormalities were identified. The most frequent chromosomal aberration found was autosomal chromosome trisomy, which accounted for 65.8% of all abnormalities. The most common identified trisomy was the trisomy of the chromosomes: 14 (14.3% trisomy), 16 (14.3% trisomy), 21 (13% trisomy), 18 (7% trisomy), and 13 (7% trisomy). In single cases, we identified trisomy of chromosomes: 3, 4, 5, 15, and 22. Our results do not differ from the results obtained by other research groups. In most studies, the trisomy rate ranged from 33 to 66%, but most often it was around 50% (Goddijn and Leschot 2000; Stephenson et al. 2002; Philipp et al. 2003; Ljunger et al. 2005). Simpson (2007) also estimated that trisomies account for approximately 50% of all identified aberrations in the material from spontaneous abortion, with the highest share of trisomy of the chromosome 16 (7.27%), 22 (2.26%), 21 (2.11%), 15 (1.68%), 13 (1.07%), 2 (1.11%), and 14 (0.82%). These 7 chromosomes account for 70% of all trisomies. The trisomies of the chromosomes 16 and 22 are the most commonly occurring in most reports (Ljunger et al. 2005). In few studies, instead of the chromosome 22, the most frequent trisomy of the chromosome 15 is reported (Stephenson et al. 2002, Philipp et al. 2003).

The second most frequently identified numerical aberration was chromosomal polyploidy (5 of 35 abnormal results). In our study, triploidy was accounted for 14.3% of all aberrations, and one case of mosaic tetraploidy with extra pair of chromosome 13. X-chromosome monosomy was observed in 2.8% (1 of 35 abnormal results). Similar results were obtained in the Phlilipp’s group - polyploidy constituted 12% of cases, but they got a much higher percentage with X monosomy, 22% (Philipp et al. 2003). According to Simpson, X-chromosome monosomy accounts for 15 to 20% of all chromosome aberrations (Simpson 2007). Based on the analysis of 11 research papers, Goddijn and Leschot (2000) found that monosomy X accounts for average 13% of all cases and ranges between individual studies from 2 to 25%. Polyploidy are usually identified in about 20% of cases with aberrations (Goddijn and Leschot 2000).

Placental mosaicism affects about 1–2% of pregnancies. Other authors show that the incidence of aneuploidy or even triploidy can be limited in several ways. The differences may concern three structures - the cytotrophoblastic layer of the chorion, the extraembryonic chorionic mesoderm and the fetal tissues themselves (Lebedev 2011). Interestingly, a complete or mosaic trisomy of the cytotrophoblast layer does not have to be associated with mesoderm or fetal tissue trisomy at the same time. Contrary to appearances, limiting the occurrence of aneuploidy only to extra-embryonic tissues (cytotrophoblast and mesoderm) with normal fetal karyotype does not mean that it does not affect the development of the embryo/fetus. In our research, the main tissue studied was the cytotrophoblastic layer of the chorion with/without the extraembryonic mesoderm layer. In our study, in one case, the presence of two cell lines was observed - normal and abnormal in the mosaic form (mosaic trisomy chromosome 14, case 1954). It is difficult to assess to what extent the mosaicism concerned only extraembryonic tissues and whether it was associated with normal/abnormal fetal karyotype or with maternal cell contamination.

Structural chromosome aberrations in our material accounted for 17.1% of identified aberrations. Our research showed a higher percentage of structural aberration detection using the aCGH method than the results of other studies. Most often, structural aberrations constitute about 5% of all identified aberrations, and according to various authors, this percentage ranges from 1.5 to 9% (Ljunger et al. 2005). Our structural variants can be divided into three groups based on the inheritance: (1) inherited from the parents, and are most likely not responsible for the miscarriage, 17p12 duplication inherited from a father with deformities of the feet and 17p13.1 deletion inherited from a healthy mother; (2)* de novo*, 1q21.1q21.2 deletion; and (3) where the origin was not specified, 3q13.31q22.2 deletion, 3q22.3q23 deletion, and 5q14.3 deletion. Among these groups, four CNVs can be responsible for the loss of pregnancy: deletion 1q21.1q21.2 (case 2205), deletion 3q13.31q22.2, deletion 3q22.3q23 (case 1545), and deletion 17p13.1 (case 2051). Another important finding is deletion 7p22.3p12.3 and duplication 9p24.3p13.2 in case number 1342 which were inherited from father with balanced translocation. Other two variants can be classified as pathogenic or likely pathogenic, however, they are rather incidental findings not correlated with the cause of miscarriage (deletion 5q14.3 and 17p12 duplication).

Rajcan-Separovic et al. (2010) were the first to carry out a study of chorion and parents to identify genomic rearrangements in couples with recurrent miscarriages, assuming that in their case, these changes should be primarily inherited, but also lead to damage to important embryonic development of genes. Their study of 27 miscarriages from 22 pairs and variant verification on 20 parents identified 11 unique CNVs inherited from mother/father, 2 of which were likely pathogenic, due to the genes present in these regions (genes *TIMP2* and *CTNNA3*) (Rajcan-Separovic et al. 2010).

Determining the parental origin of the aberration found in the fetus is extremely important not only for determining the prognosis for the maintenance of subsequent pregnancies, but also for proper genetic counseling. One of the most common causes of miscarriages, infertility or congenital fetal defects is the carrier of the balanced and Robertsonian translocation. The chance, that one of the parent is a carrier of balanced translocation increases with the number of miscarriages (Goddijn et al. 2004). However, as previously shown, in the case of the carrier, only about 30% of aborted embryos/fetuses inherit an unbalanced translocation, another 30% have a normal or balanced karyotype, and in the other cases, aneuploidy of the autosomal chromosome was identified (Carp et al. 2006; Stephenson and Sierra 2006; Simpson 2007).

In our study, in one case, aCGH revealed unbalanced changes which were derivative of parental balanced translocation. We detected the presence of 7p22.3p12.3 deletion and 9p24.3p13.2 duplication (case 1342). Taking into account the size of these aberrations (45.74 Mb and 38.14 Mb), these were classified as pathogenic aberration responsible for miscarriage. The karyotype of the parents showed that aberrations observed in the fetus were a derivative of paternal balanced translocation. Due to the lack of research on previous failures, it cannot be unequivocally concluded that in the remaining cases, the cause was the same. Determining the origin of the identified aberrations gives this couple a chance to have healthy offspring thanks to the currently widely used method of preimplantation genetic testing of structural rearrangements (PGT-SR). The incidence of balanced structural aberrations in reproductive failure couples ranges from 2 to 6% (De la Fuente-Cortes et al. 2009; Karim et al. 2017). Interestingly, study conducted by Lovrečić et al. (2019) suggested that in some families, translocation is inherited in a balanced form and the occurrence of pregnancy failure may be related to other non-genetic factors.

The aCGH method, in addition to detecting large structural imbalances, can in particular identify submicroscopic genome imbalances (Dhillon et al. 2014). However, despite the fact that different de novo CNVs and those inherited from parents are found, their contribution to developmental failure is largely unknown (Rajcan-Separovic et al. 2010; Wang et al. 2017). It is estimated that the incidence of aberrations of unknown clinical significance (VOUS) in spontaneous miscarriages is approximately 2% (Dhillon et al. 2014).

It is well known that in any diagnostic study “incidental findings” can also be found. A prime example is the ~ 142 kb deletion of the 5q14.3 region (case number 2193) found in our study. Aberration involves exons 22–74 of the *GPR98* gene (OMIM: 605472). Mutations in the *GPR98* gene have been described in patients with Usher syndrome type IIC. Unfortunately, due to the lack of contact with the parents, the origin of the aberration was not specified. Usher syndrome is an autosomal recessive disorder. It is mainly characterized by sensorineural hearing loss and retinitis pigmentosa. It is the most frequent cause of combined deafness and blindness in adults and affects 3 to 6% of children born with hearing impairment. We did not find a similar deletion in the ClinGen, DECIPHER database, or Database of Genomic Variants. So far, no association has been found between this gene and pregnancy failure, or any possible effect on ovarian function.

The presence of an aberration inherited from the parent does not exclude a mutation on the second allele causing an abnormal phenotype and/or fetal death. A rare aberration found in our study is the deletion of the short arm of chromosome 17 in the 17p13.1 region with a size of ~ 30.25 kb (case number 2051). The aberration involves exons 6–40 of the dose-sensitive *MYH8* gene (OMIM: 160741). As in the previous case, we did not identify a similar deletion in the ClinGen, DECIPHER database, or Database of Genomic Variants. The *MYH8* gene encodes the heavy chain of myosin 8 involved in fetal skeletal muscle development. Parental aCGH studies have shown that the 17p13.1 deletion found in the fetus is inherited from healthy mother. This patient is chronically ill with Hashimoto’s disease and has been diagnosed with the presence of the left kidney double system. Myosin heavy chain 8 (MYH8) is an ATP-dependent motor myosin that is predominantly expressed in prenatal muscle and involved in muscle development. Myogenic factors are certainly necessary for normal fetus development and may also play a role in neurogenesis (Kablar et al. 2003). Their findings also suggest that this embryonic isoform myosin component plays a role in brain development. Mutations in the *MYH8* gene are described in the Trismus-pseudocamptodactyly syndrome with autosomal dominant inheritance (OMIM: 158300). Lou et al. (2020) examined 152 Chinese patients with ovarian endometriosis. In two patients, they demonstrated the presence of two heterozygous missense mutations in the *MYH8* gene: c.1441A > C (p.I481L) (34-year-old patient) and c.4057G > A (p.E1353K) (25-year-old patient). These mutations have not been found in public databases and not have been detected in a control group of 485 Chinese women without endometriosis. They concluded that mutations of the *MYH8* gene can play a role in the pathogenesis of endometriosis. Women with endometriosis are at high risk of early miscarriage, ectopic pregnancy and premature delivery, and postpartum hemorrhage. Therefore, we classified this aberration (deletion 17p13.1) as likely pathogenic.

In another case (case number 2205), we found 1.3 Mb deletion of chromosome 1q21.1q21.2. The test result was confirmed by the MLPA method (SALSA MLPA probemix P297-B2). Parental MLPA studies showed that the aberration arise de novo. The deletion is located in the region of the known 1q21.1 microdeletion syndrome (OMIM: 612474). A similar deletion was also reported by Gamba et al. (2016) who described one Brazilian patient with interstitial microdeletion and indicated that this deletion may be responsible for the spontaneous miscarriage.

Recent reports show that mutations in genes responsible for the proper fetal development are a more frequent cause of reproductive failures than chromosomal aberrations. Analyzing the pedigrees of families affected by spontaneous abortions, it can be stated that in the genome, there are many genes whose functions are not associated with pregnancy loss and the research conducted on mouse models indicate that mutations in these genes may be responsible for the failure of reproduction (Colley et al. 2019). One such example is the *EGFR* gene which is a growth factor produced by epithelial cells and is the most important growth regulator in these cells. Dackor et al. (2007) in their research found that the *EGFR* gene shows high expression in murine placenta cells and participates in the process of trophoblast cell proliferation. Homozygous mutations in the *EGFR* gene lead to underdevelopment of placental cells and death of embryos (Dackor et al. 2007). Advances in sequencing technology, including whole exome sequencing (WES) and whole genome sequencing (WGS), are increasingly making it possible to detect genetic sequence variation and characterize the genetic mutations that cause disease. The first studies using WES in pregnancy structural disorders, late miscarriages, and fetal development disorders were described by Hillman et al. (2015) and Shamseldin et al. (2018). However, the number of families with recurrent miscarriages studied so far is still too small to indicate a complete list of mutations or genes responsible for this process. The available literature contains descriptions of biological processes, the pathogenesis of which may contribute to the death of fetus. Examples include mutations in genes *RYR1* and *GLE1* found in the fetal akinesia deformation sequence (FADS) or the *KIF14* gene involved in ciliary function and cell division (Rajcan-Separovic 2020).

The results of subsequent WES tests contribute to the creation of a comprehensive database containing information on mutations contributing to fetal death and the inheritance of these changes. This will enable a broader understanding of the causes of recurrent miscarriages and the development of strategies that lead to a successful pregnancy for couples with recurrent miscarriages.

Our research has proven that the method of comparative genomic hybridization to microarray is effective in identifying the most common genetic aberrations, submicroscopic genomic rearrangements, as well as genes whose mutations contribute to miscarriages. Of the 62-examined trophoblast/fetal skin fibroblasts, we found chromosomal aberrations in 56.5% cases, and 82.9% of abnormal results were chromosomal numerical aberrations (trisomies, X-chromosome monosomy and polyploidy), but as many as 17.1% were small aberrations that cannot be identified by the classical method GTG. This certainly shows the enormous potential of this method in identifying of all aberrations that were previously beyond the reach of available research methods. It is also a chance for couples with normal karyotype of both partners and embryo/fetus to identify the cause of miscarriages, especially recurrent ones. This method also makes it possible to find regions where are located important genes for embryonic growth, and damage to which may lead to pregnancy failure. On the other hand, an additional chance to find out the causes of spontaneous miscarriages is created by the WES method, widely used in recent years.
